# Potential use of the Bushmint, *Hyptis suaveolens*, for the Control of Infestation by the Pink Stalk Borer, *Sesamia calamistis* on Maize in Southern Benin, West Africa

**DOI:** 10.1673/031.011.0133

**Published:** 2011-03-22

**Authors:** Cyrille Adda, Pierre Atachi, Kerstin Hell, Manuele Tamò

**Affiliations:** ^1^ Africa Rice Center (AfricaRice), 01 B.P. 2031, Cotonou, Benin, West Africa; ^2^ Université d'Abomey-Calavi, Faculté des Sciences Agronomiques (FSA/UAC) 01 B.P. 526, Cotonou, Bénin, West Africa; ^3^ International Potato Center (CIP), 01 B.P. 2031, Cotonou, Benin; ^4^ International Institute of Tropical Agriculture, 08 B.P. 0932, Cotonou, Benin, West Africa

**Keywords:** aqueous extract

## Abstract

Maize production in Benin, especially in resource-poor farmers' fields, is constrained by stemborers among other factors. One of the major stemborers in southern Benin is *Sesamia calamistis* Hampson (Lepidoptera: Noctuidae). African farmers cannot afford to use commercial insecticides for controlling stemborers - they are expensive and unsuitable for durable pest management systems due to eco-toxicity. There is therefore a need for cheaper and environmentally friendly methods and botanicals offer an attractive alternative. The bushmint, *Hyptis suaveolens* (L.) Poit. (Lamiales: Lamiaceae), was compared with the commercial insecticide Furadan (carbofuran) for the control of *S. calamistis* on maize *Zea mays* L. (Poales: Poaceae). Trials were conducted in the screenhouse and in the field during the minor cropping season in 2004 at the International Institute of Tropical Agriculture (IITA)-Benin station. The variables measured included numbers of egg masses per plant, eggs per egg mass (in the screenhouse study), population density of *S. calamistis*, percentage of infested plants and/or ears, and deadhearts in the field. Irrespective of the variable considered, the aqueous extract of *H. suaveolens* compared favorably with Furadan while maize surrounded by live *H. suaveolens* plants had lower *S. calamistis* densities.

## Introduction

Maize, *Zea mays* L. (Poales: Poaceae), has become one of the most important staple foods in sub-Saharan Africa (World Bank 1997; FAO 2007). It is an integral part of the social and economic life of the people (Adandé 1984; Lutz et al. 1995) with a per capita consumption of over 100 kg per year especially in the central and southern regions of the country (ONASA 1997).

Maize production in Benin is constrained by insect pests, among which stemborers are the most important. The larvae of several Lepidoptera inflict serious damage on pre-harvest maize stems and ears and significantly lower maize yields (Atachi 1984, 1987; Bosque-Pérez and Mareck 1990; Schulthess et al. 1991). Yield reduction due to stemborers ranges from 20% to total crop failure (Shanower et al. 1991; Bosque-Perez and Schulthess 1998). Of the ten identified species of maize Lepidoptera in Benin, the most frequently encountered are *Sesamia calamistis* Hampson (Lepidoptera: Noctuidae), *S. botanephaga, Eldana saccharina, Busseola fusca; Mussidia nigrivenella, Chilo* spp.; *Cryptophlebia leucotreta*, and *Spodoptera exempta* (Atachi 1987; Shanower et al. 1991). *Sesamia calamistis* is a serious pest, especially on maize in the minor season in southern and central Benin (Atachi 1987) and on late maize in the northern regions.

The use of chemical pesticides against stemborers is limited not only because of their high costs (generally not affordable for small farmers) and scarce availability in rural areas, but also due to the health and environment concerns related to them. There is a need for cheaper and safer alternative control practices. Over the years, farmers have learned to contain pest problems through the use of plant extracts. The potential advantages of botanical pesticides over synthetic pesticides have been highlighted by Prakash et al. 2008. Many plants have been tested or identified as interesting botanical pesticides in sub-Saharan Africa (Kossou et al. 2001a, b; Oparaeke et al. 2005; Sinzogan et al. 2006; Oparaeke et al. 2006a, b; IITA 2006; Okereke et al. 2007; Oigiangbe et al. 2007; Aboubakary et al. 2008) and are potentially usable in pest control programs taking into account both the needs of increased food and preserving the health of a growing population.

In Benin, only a limited database is available on the use of botanicals to control insect pests on crops in the field. Some attempts made in the past to reduce pest pressure on crops using botanicals focused on legumes, mainly cowpea (PRONAF 2000; Kossou et al. 2001a, b; James et al. 2003; Gbehounou 2007). Furthermore, neem (*Azadirachta indica*) oil was successfully used to control stem borers on maize in the field (Bruce et al. 2004). Also, recent research findings revealed that a combination of the aqueous extract of the bushmint *Hyptis suaveolens* (L.) Poit. (Lamiales: Lamiaceae) with a lower dose of insecticides such as Thionex 350 EC (Endosulfan (350)) or Laser 480 EC (Spinosad (48)) helped to successfully control cotton bollworms (Sinzogan et al. 2006)

*Hyptis suaveolens* is used for some ethnobotanicals applications in rural communities in African countries (Koumaglo et al. 1993; Kossou et al. 2001a, b; Edeoga et al. 2006) and the plant is readily available close to villages, along roadsides, on farmsteads, etc. The objective of this study was to evaluate the potential use of *H. suaveolens* plants for the control of *S. calamistis* on maize.

## Materials and Methods

### Study locations

Field and screenhouse experiments were conducted at the Research Station of IITA in Abomey-Calavi [15 m altitude, latitude 6° 25′ E and longitude 2° 20′ N]. The site is located in the Coastal Savannah characterized by a bimodal rainfall with two peaks, one in June and another in October, and an annual rainfall of 1100 – 1500 mm. The first and main production season extends from April to July while the minor season covers the months of September to November. The long dry season starts in November and lasts until March. The average annual temperature fluctuates between 25 and 30° C with a minimum in July and August and a peak in March. The field trial was run in the minor cropping season in 2004, which was characterized by high precipitation especially in September (356.2 mm) and October (219.1 mm). The monthly average temperature fluctuated between 25.2° C and 26.8° C and the average monthly relative humidity ranged from 81.2 to 87.7%. The trial was conducted in the field on a sandy-clay soil. The screenhouse had a temperature of 25 to 28° C and relative humidity of 75 to 80%.

### Experiment 1: Screenhouse study

**Potted maize production and use.** The improved, early maturing (90 days) IITA maize variety DMR, resistant to downy mildew, *Peronosclerospora sorghii*, and maize streak virus, was used for this experiment. The average yield of DMR is 4 metric tons ha^-1^ with a potential of 7 to 8 metric tons ha^-1^ in highly fertile soils with low pest pressure (Bosque-Pérez and Mareck 1989). Maize grains were sown in black plastic pots (30 cm in diameter and 40 cm in height) filled with soil in the field and the seedlings were thinned to 1 plant per pot. The pots received NPK fertilizer (14–23–14) at the rate of 3 g per pot. Three weeks after planting, the pots were transferred to the screenhouse where they acclimatized for 5 days before the release of adults of *S. calamistis*. This procedure ensured that any eggs deposited on the plants while in the field were not taken into account when counting eggs five days after the release of adults in the screenhouse.

**Cultivation of *H. suaveolens* and preparation of the aqueous extracts.** Three to four-week-old *H. suaveolens* seedlings were transplanted onto a 0.8 ha plot at the IITA experimental site and watered regularly until the onset of the rains. One month after transplanting, fresh leaves and stems were weekly collected for the preparation of the aqueous extracts.

The procedure for obtaining the aqueous extract of *H. suaveolens*, the concentrations of extract per hectare, and the dose administered were based on the technology developed by the Cowpea Project for Africa, whereby 37.5 kg of fresh *H. suaveolens* were used to treat one hectare of cowpea (PRONAF 2000). Following the same approach leaves and stems of *H. suaveolens* were collected at sunset and crushed in a mortar until soft dough that was subsequently transferred into a bowl. The rest of the herbal substance was recovered by rinsing the mortar. Some 300g of powder soap were added to the mixture that was stirred and left to rest overnight. The subsequent morning the mixture was filtered with a fine tissue, and water was added to the filtrate to obtain a concentration of 10:100 (weight: volume). Then 300 ml of kerosene were added and the resulting solution was vigorously stirred. The soap acted as an adjuvant enhancing the adhesion of the extract on the surface of treated plants while the kerosene was used to prevent soap bubbles. In the current investigation, various concentrations of *H. suaveolens* were tested in the screenhouse (Table 1) to identify the best concentration for reducing the incidence of *S. calamistis* on maize.

**Production of larvae and adults of *S. calamistis*.** Virgin adults of *S*. *calamistis* used in the screenhouse were obtained from the mass rearing culture maintained over several generations at IITA-Benin. The insects were reared in a room maintained at a temperature of 25 to 27° C, 75 to 80% relative humidity, and 14:10 (L:D) illumination. Insects were sexed at the nymphal stage. The eggs were incubated until the blackhead stage, and then transferred to an artificial medium. The medium was originally developed at ICIPE (Onyango and Ochieng-Odero 1994) for *B. fusca*, but proved to be suitable for the breeding of many other borers including *S. calamistis* (Songa et al. 2001).

**Experimental design of the screenhouse study.** A randomized complete block design with five treatments replicated eight times was used. The treatments were individual potted maize plants treated with various concentrations (C) of *H. suaveolens* extract (Table 1) as follows:

Treatment 1: No plant extract; 0g L^-1^ (C1 = 0%);
Treatment 2: Plant treated with extract at a dose of 50g L^-1^ (C2 = 5%)
Treatment 3: Plant treated with extract at a dose of 100g L^-1^ (C3 = 10%)
Treatment 4: Plant treated with extract at a dose of 150g L^-1^ (C4 =15%)
Treatment 5: Plant treated with extract at a dose of 200g L^-1^ (C5 = 20%)

**Table 1.**
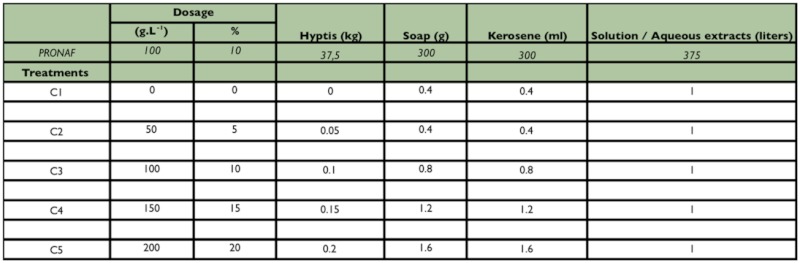
Preparation of different doses of aqueous extracts of *H. suaveolens* for screenhouse study

The quantities of *H. suaveolens* and other ingredients (kerosene and soap used to improve the adherence of the extract to the maize plants) are given in Table 1. The proportions of these ingredients were equal to those proposed by PRONAF (2000). A fixed quantity of 1 liter of each concentration of the aqueous extract was prepared and partially used to treat the eight plants receiving the same concentration in each trial. The spraying took place the same day prior to release of adult insects. All parts of the plants were copiously wetted with the extract using a local hand-held mist blower and the remaining quantity of each concentration was discarded. Within the screenhouse, the blocks (or repetitions) were 2 m apart and separated by mosquito nets. Within a block, the plants treated differently were also separated by 2 m. Four pairs of virgin adults were released into each block (making a total of 32 couples of insect for the screenhouse) and, after five days, the number of egg masses per plant and number of eggs per mass were recorded. The trial was set up four times.

### Experiment 2: Field trial

**Field maintenance and fertilizer application.** The field was planted on 27 August 2004 at IITA, using the same maize variety (DMR). Two weeks after sowing, maize was thinned to one seedling per hill at a spacing of 80 cm between rows and 25 cm within rows, giving a plant density of 50,000 plants ha^-1^. The plots received NPK fertilizer (14–23–14) at a dose of 150 kg ha^-1^ 3 weeks after planting and 100 kg ha^-1^ of urea 6 weeks after planting. All plots were weeded at least twice before harvest, and generally prior to the application of fertilizer.

**Experimental setup of the field trial.** The randomized complete block design with four treatments replicated three times was used. The following treatments were allocated to 10 m × 5 m (50 m^2^) plots:

Treatment 1 (T1): sole crop maize without protection (no *H. suaveolens* and no insecticide).
Treatment 2 (T2): maize plot surrounded by 2 strips making a total of approximately 250 plants of *H. suaveolens*.
Treatment 3 (T3): maize plot treated with an aqueous extract of *H. suaveolens* at a dose of 75 kg in 375 liters of extract per hectare (approximately 2 liters of aqueous extract of 200 g L^-1^ (20%) per 50 m^2^ experimental plot).
Treatment 4 (T4): maize plot treated with granular Furadan (carbofuran) (applied in the cone leaf of the plant 3 weeks after planting at a rate of 2 g per plant).

The three replicates were actually three blocks of 39 m × 14 m each, plowed to a depth of 20 cm with a tractor, and separated by 150–200 m to reduce interaction between treatments.

In T2, seedlings of *H. suaveolens* (30 to 40 cm tall), were transplanted at a density of 200 to 250 seedlings per plot 10 weeks before maize planting. In treatment 3 (T3), the maize plants were carefully sprayed every week, from the third to the twelfth weeks after planting with an aqueous extract of *H. suaveolens* prepared on the eve of the spraying performed using a 15 L sprayer (Cooper Pelger, www.cooper-pegler.com). Whenever it rained the day of spraying, the operation was repeated the next day.

**Sampling method and parameters measured.** Destructive sampling was done weekly from 3 to 12 weeks after planting according to Atachi (1984). Samples of 10 randomly chosen maize plants were collected along the diagonals and medians of the plots and at calibrated distances. Seedlings were pulled by hand while older plants were cut with a knife just above the collar and then dissected. The dissected samples were inspected in the field if infestation was low, or taken to the laboratory where the following parameters were recorded: number of *S. calamistis* (larvae or pupae), percentage of plants infested by *S. calamistis*, percentage of dead hearts, and percentage of ear infested by *S. calamistis*.

**Data analysis.** For the screenhouse study, a univariate analysis of variance was performed using SAS software (SAS Institute Inc. 2001) to compare the effect of different concentrations of the aqueous extract of *H. suaveolens* based on the numbers of *S. calamistis* egg masses per plant and eggs per mass. Statistical analysis of field data was preceded by the normalization of variances. Data on *S. calamistis* density were log-transformed and the numbers of dead hearts or ears infested by *S. calamistis* were square root-transformed. An analysis of variance on repeated measures of different variables was made using SAS software (SAS Institute Inc. 2001). Means were separated for significance using the Student Newman-Keuls (SNK) test.

## Results

### Experiment 1: Screenhouse study

**Effect of *H. suaveolens* aqueous extract on the number of *S. calamistis* egg masses.** The number of egg masses per plant decreased linearly with an increase in the concentration of the aqueous extract (Table 2A). Highly significant differences were observed between treatments (F = 28.334, DF = 4, P = 0.000). The SNK test showed that there were significantly more egg masses on the control (T1) than on all treated plants. Treatments T4 and T5 (concentrations 15% and 20%) gave statistically similar results, but were significantly better than treatments T2 and T3 (concentrations 5% and 10%), which were also significantly different from each other (Table 2A). Compared with the untreated control, the aqueous extract of *H. suaveolens* reduced the number of egg masses per maize plant by almost 50%.

**Table 2.**
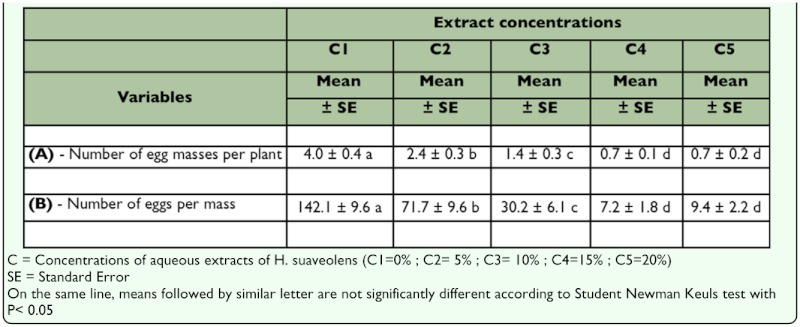
Comparison of treatments (Means ± Standard Errors) based on the numbers of egg masses and eggs per mass laid by *Sesamia calamistis* on maize plants (N= 32 maize plants for each treatment)

**Effect of *H. suaveolens* aqueous extract on the number of *S. calamistis* eggs per mass.** There was a linear decrease in the number of eggs per mass with an increase in the concentration of the aqueous extract (Table 2B). Highly significant differences were found between treatments (F = 35.277, DF = 4,P = 0.000). The SNK test indicated that significantly higher numbers of eggs per mass were laid on plants in the control (T1) than on all treated plants. Treatments T4 and T5 (concentrations 15% and 20%) were statistically identical but significantly better than treatments T2 and T3 (concentrations 5% and 10%), which were also significantly different from each other (Table 2B). In comparison with the untreated control, the aqueous extract of *H. suaveolens* reduced the number of eggs per mass by at least half.

### Experiment 2: Field study

**Effect of treatments on the infestation of maize plants by *S. calamistis*.** The treatments significantly affected the percentage of plants infested by *S. calamistis* (F_3, 70_ = 48.94, P <0.0001) (Table 3A). However, their interaction with the sampling dates was not significant (F_8, 70_ = 5.20, P = 0.3130) (Table 3A). The SNK test showed that all protection measures tested (T2, T3, and T4) were significantly different from the control (T1). Furadan (T4) had statistically the same protective effects as the aqueous extract of *H. suaveolens* (T3), but was significantly more effective than T2 (*H. suaveolens* plants surrounding the maize plot). There was no significant difference between the two treatments that included the use of *H. suaveolens* (whether as a vegetative association with maize (T2), or as a 20 % aqueous extract (T3)) (Table 4A). The use of *H. suaveolens* in a vegetative association with maize in the field reduced by half the percentage of plants infested by *S. calamistis* compared to the control. Furthermore, maize plant infestation by *S. calamistis* was more than three times higher in the control than in maize plots treated with the 20 % aqueous extract of *H. suaveolens* (Table 4 A).

**Table 3.**
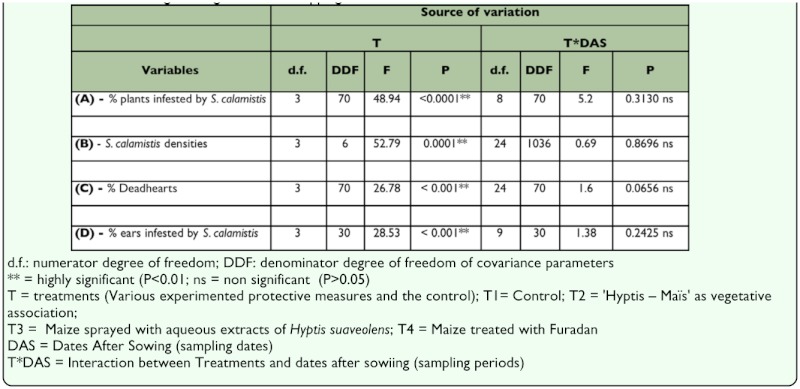
Repeated measure analysis of variance of % infested plants, *Sesamia calamistis* densities per plant, % Deadhearts, % infested ear and ear damage during the minor cropping season in 2004 in southern Benin

**Effect of treatments on *S. calamistis* densities.** The treatments significantly affected *S. calamistis* densities (F_3,6_ = 52.79, P = 0.0001) (Table 3B). Due to the non-significant interaction between treatments and sampling periods (F_24, 1036_ = 0.69, P = 0.8696), means for all sampling periods were pooled. The SNK test showed that there were significantly more *S. calamistis* in the control than in all other treatments (T2, T3 and T4). The vegetative association of maize with *H. suaveolens* (T2) was significantly less effective than T3 (aqueous extract of *H. suaveolens*) and T4 (Furadan). No significant differences existed between T3 and T4 (Table 4B). The mean density of *S. calamistis* in the control (T1) was nearly three times that in T2 and 10 times that in T3. Plots treated with Furadan (T4) had the lowest density of the insect but this was not statistically different from using *H. suaveolens* aqueous extract (T3) (Table 4B).

**Effect of treatments on percentage of deadhearts.** The treatments also significantly affected the mean percentage of deadhearts (F_3, 70_ = 26.78; P < 0.0001) (Table 3C). Due to the non-significant interaction between treatments and sampling periods (F_24, 70_ = 1.60; P = 0.0656), the comparison of means for all sampling periods was combined. The SNK test showed that all the protective measures (T2, T3, and T4) were significantly different from the control (T1). The mean percentage of deadhearts in the control was more than double the percentage found in T2. The extract of *H. suaveolens* (T3) performed better than the vegetative association of maize with *H. suaveolens* (T2) (1 versus 5 deadhearts per 100 plants) and gave statistically similar results with Furadan (T4) (Table 4C).

**Table 4.**
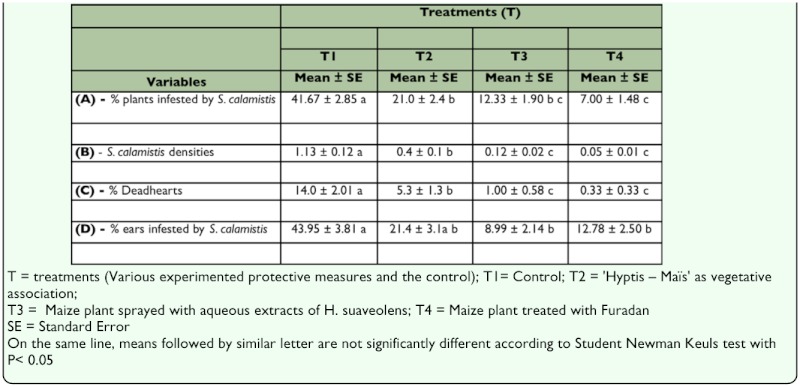
Comparison of treatments (T1, T2, T3 and T4) based on *%* infested plants, *Sesamia calamistis* densities per plants, *%* Deadhearts, *%* ears infested and cob damage during the minor cropping season in 2004 in southern Benin

**Effect of treatments on the percentage of infested ears.** The treatments significantly affected the average percentage of ears infested by *S. calamistis* (F_3, 30_ = 28.53, P <0.0001) (Table 3D), but their interaction with the sampling periods was not significant (F_9, 30_ = 1.38; P = 0.2425). The SNK test demonstrated that only treatments T3 and T4, which were statistically identical, were significantly better than the control T1. The vegetative association of maize and *H. suaveolens* (T2) did not reduce stemborer infestation of the ears compared to the control T1 (Table 4D).

## Discussion

The results of the screenhouse study showed that the aqueous extract of *H. suaveolens* adversely affected egg production by *S. calamistis*, reducing by at least half the number of egg masses per maize plant as well as the number of eggs per mass compared to the control. Based on these two parameters all the treatments performed better than the untreated control. The reduction in egg production on the treated plants may be due to probable oviposition deterrent properties of the extracts and confirms previous results on lepidopteran pests (Raja et al. 2005). The oviposition deterrent activity of the aqueous extract of *H. suaveolens* that prevented the borer from laying eggs on the maize plants in the screenhouse seemed to be confirmed in the field experiment. In fact, significantly higher densities of *S. calamistis* were recorded in the control than on plants treated with *H. suaveolens* extracts. The percentage of maize plants infested by *S. calamistis* was more than three times higher in the control than in maize plots treated with the aqueous extract of *H. suaveolens*. The same was true for other parameters (percentage of infested ears and deadhearts).

The spraying of the aqueous extract of *H. suaveolens* on maize plants at weekly intervals, may have acted as an inhibitor that stopped the development of different stages of *S*. *calamistis. Hyptis suaveolens* has been reported to be biologically effective against lepidopteran pests (Prakash et al. 2008) and may have caused insects feeding on the maize plants to reduce their food intake. The results of the current studies are in accordance with previous studies reporting the efficacy of *H. suaveolens* on various pests infesting and damaging diverse crops. Extracts of *H. suaveolens* were found to possess significant ovicidal and antifeedant activity against *Helicoverpa armigera* on cotton (Raja et al. 2005). A leaf extract of *H. suaveolens* reduced the population of *Spodoptera litura* and *Aphis craccivora* on groundnut (Ignacimuthu and Jayaraj 2003; Jayakumar et al. 2004). On cowpea, various solvent extracts of *H. suaveolens* were found to be efficient in terms of their oviposition deterrent, ovicidal, or insecticidal effects against *Callosobruchus maculatus* (Jayakumar et al.2005). Oparaeke et al. (2005) found that a combination of *H. suaveolens* extracts and neem extracts greatly reduced pod damage by *Maruca vitrata* and *Clavigralla tomentosicollis*, and produced more yield than the unsprayed control. Extracts of *H. suaveolens* have been successfully used not only against devastating insects, but have also contributed to the reduction of *Sclerotium* wilt of tomato caused by *Sclerotium rolfsii* by exerting some anti-fungal protective action on the tomato plants (Okereke et al. 2007).

The results of this study also showed that intercropping *H. suaveolens* with maize can reduce attack by *S. calamistis* on maize in the field. The vegetative association of *H. suaveolens* with maize contributed to the reduction of *S. calamistis* infestation and densities of the ear borer per plant. The idea that *H. suaveolens* plants may have driven away the adult *S. calamistis* from the maize plant by their smell is not as convincing as the presumption that the plant probably played a disturbing role. Vandermeer (1989) listed three possible mechanisms responsible for reduced pest infestation in mixed cropping systems: (a) the disruptive-crop hypothesis, in which a second non-host plant species disrupts the ability of the pest to find its proper host plant species; (b) the trap crop hypothesis in which a second non-suitable host plant species attracts the pest away from its primary host; (c) the natural enemy hypothesis in which the intercropping situation attracts more predators and parasitoids than the monocrop, thereby reducing pests on the primary host plant. *H. suaveolens* may have played the role of a disruptive plant reducing both chemical and visual cues and stimuli of *S. calamistis*, thus interfering with its ability to encounter maize plants.

*H. suaveolens* extract compares favorably with the insecticide Furadan in reducing *S. calamistis* densities on maize. In a previous study, *H. suaveolens* leaf extract also gave similar effect in comparison with the fungicide Captan on *Sclerotium* wilt of tomato (Okereke et al. 2007). Many other plant extracts have exhibited insecticidal, ovicidal, antifeedant, and oviposition deterrent activity against lepidopteran pests (Juan et al. 2000; Arnubio et al. 2006; Oigiangbe et al. 2007; Prakash et al. 2008; Aboubakary et al. 2008). Botanical insecticides have long been touted as attractive alternatives to synthetic chemical insecticides for pest management because they reputedly pose little threat to the environment or human health. Since there are variations in the chemical composition of *H. suaveolens* from different locations (Peerzada 1997; Azevedo 2001), further work should include scaling up of the current results through screening of this plant and evaluation of other plant extracts for the control of the maize stemborer and other insect pests so as to increase the control options immediately available to farmers.

The current studies revealed that in the context of integrated pest management, the aqueous extract of *H. suaveolens* may play a significant role in pre-harvest maize protection against stemborers. Nevertheless, the preparation of the extract is laborious and time consuming. Also the quantity used is rather large and would be one of the constraints for using aqueous extracts. All this calls for innovative methods and more appropriate formulation, perhaps in the form of essential oils. In spite of the promising results obtained with the vegetative association of *H. suaveolens* with maize, this technique needs to be considered with caution. *H. suaveolens* is a fairly prolific plant with luxuriant growth, forming dense stands within a few weeks and it might compete with crops for space, water, and nutrients. The plant is neither reported as food nor as feed in Benin, so it does not possess any intrinsic added value to the cropping system apart from deterring insect pests.

## Abbreviations

**DMR**,: downy mildew resistant
**ICIPE**,: International Center of Insect Physiology and Ecology
**IITA**,: International Institute of Tropical Agriculture
**SNK**,: Student Newman-Keuls
